# Animalcules in the Oil-tubes of the Skin

**Published:** 1846-04

**Authors:** 


*Animalcules in the Oil-Tubes of the Skin.*—The following information may possibly be new to some of our readers:

"The animalcule of the skin," says Mr. Wilson, "is found in the oil-tubes, whenever there exists any disposition to the unnatural accumulation of their contents; it is found in number varying from one or two to twenty, in the interior of the little grooved cylinder, which is squeezed out by pressure of the fingers; and this in an apparently perfect state of the health of the skin, if it can be said to be in health when its functions are performed in a torpid manner. Now, as in the majority of mankind, and certainly in all the inhabitants of cities and large towns, the skin is more or less torpid in its functions, so the presence of this animal in the skin is the rule, its absence the exception; I have found it in all ages, from youth to old age, more numerously, it is true, at the latter than at the former period, and in great and remarkable numbers during sickness. Under these circumstances I see no other conclusion open, than to assume that it performs some beneficent purpose in the economy of the skin, that purpose being, according to my belief, the disintegration of the over-distended cells, and the stimulation of their tubes to perform their office more efficiently. In corroboration of this view, is the fact, that these little creatures increase in number when the vital powers decline, so that when the energies of the system are reduced by disease, and when the skin, participating in the reduction, is unable alone to fulfil its functions correctly, these little beings are produced to aid in the work."

The British and Foreign Review objects to the name *Entozoon Folliculorum*, by which Mr. Wilson designates this animal, and says that "it unites the characters of the higher annelida, or worm-tribe of the lower arachnida or spidertribe, and of the lower crustacea or crab-tribe, to such a degree, and it is yet doubtful which class ought to afford it a place. The investigator of the history of this parasite need certainly be in no lack of subjects for examination, for, strange to say, it is one of the most readily procurable of all living beings, especially in a town-population. Here, as elsewhere," continues the writer, "we see how nature manages to compensate one class for the exemption they may enjoy from the evils incident to another. The delicate town-bred lady of fashion, in ascending from her carriage, shrinks instinctively from the mass of rags, filth, and vermin, which is brought into contiguity with her precious person by some pertinacious beggar, ignorant all the while that her sebacious follicles give board and lodging to a host of parasites, whose number may equal that of the various kinds of 'small deer' that nestle in the matted hair and tattered garments of the fellow-being whom she regards with so much loathing.—'Where ignorance is bliss, 'tis folly to be wise.' "—*Ibid.*

